# Veterinary Department, Ohio State University

**Published:** 1895-07

**Authors:** 


### AMONG THE COLLEGES.

### Veterinary Department, Ohio State University.

No more painful news has come to us, as editors and willing promoters of every movement to advance the standard of veterinary education in America and to aid and succor the interests of every veterinary school whose aims and purposes are directed toward the achievement of higher education, than the announcement at this time that the Veterinary Department of the Ohio State University at Columbus, Ohio, has closed.

Established almost wholly through the untiring efforts of Prof. H. J. Detmers, upon a broad scale, a thorough curriculum, and gaining year by year a strong position, a well-recognized place, and a future destiny that was to redound to the honor and glory of those whose whole energy and interest were wrapt up in its work—equally as high an honor to the profession—we regret exceedingly the unfortunate circumstances that led to its closure.

Most of all are we pained to learn that the question of its expense as a department was one of the chief causes that loses to the profession this well-founded institute of learning, because no State could be said to have greater interests at stake in the live-stock industry than Ohio, and no school was destined to turn out a better corps of men calculated to solve these all-important questions for her people than those schooled at this institution.

Those who had the pleasure of meeting Prof. Detmers at the Congress of Faculties of the Veterinary Colleges of North America will recall his earnest appeal and efforts for a high minimum standard, and when we subsequently learned that one of the chief veterinary instructors was abroad, better fitting himself for more thorough laboratory and bacteriological work, we were filled with rejoicing at the promise of another school whose work was to be of such a high character that it would strengthen the hands of the National Association in leading higher veterinary education in America.

We sincerely sympathize with Prof. Detmers, whose life-work and future were so completely wedded to this work, and we sincerely regret that its closing will cost the veterinary profession as teachers the loss of Professors Paul Fischer and D. S. White, the former we understand having already accepted a much more lucrative position in connection with the University of Utah; while the latter will perhaps be associated in bacteriological work in the Sheffield School of Science at Yale University.

After ten years of faithful work in establishing the Veterinary Department, we understand that the purpose is to strengthen the departments of Law, Pottery, and Astronomy. We were not aware that these branches of learning so far outranked in importance in Ohio those so much more closely allied to the chief interests of her people, viz., agriculture.

